# Phenotypic effects of dietary stress in combination with a respiratory chain bypass in mice

**DOI:** 10.14814/phy2.14159

**Published:** 2019-07-02

**Authors:** Praveen K. Dhandapani, Annina M. Lyyski, Lars Paulin, Nahid A. Khan, Anu Suomalainen, Petri Auvinen, Eric Dufour, Marten Szibor, Howard T. Jacobs

**Affiliations:** ^1^ Faculty of Medicine and Health Technology Tampere University Tampere Finland; ^2^ Institute of Biotechnology University of Helsinki Helsinki Finland; ^3^ Stem Cells and Metabolism Research Program, Faculty of Medicine University of Helsinki Helsinki Finland

**Keywords:** mitochondria, obesity, respiratory chain, high‐fat diet, ketogenic diet, microbiome

## Abstract

The alternative oxidase (AOX) from *Ciona intestinalis* was previously shown to be expressible in mice and to cause no physiological disturbance under unstressed conditions. Because AOX is known to become activated under some metabolic stress conditions, resulting in altered energy balance, we studied its effects in mice subjected to dietary stress. Wild‐type mice (*Mus musculus*, strain C57BL/6JOlaHsd) fed a high‐fat or ketogenic (high‐fat, low‐carbohydrate) diet show weight gain with increased fat mass, as well as loss of performance, compared with chow‐fed animals. Unexpectedly, AOX‐expressing mice fed on these metabolically stressful, fat‐rich diets showed almost indistinguishable patterns of weight gain and altered body composition as control animals. Cardiac performance was impaired to a similar extent by ketogenic diet in AOX mice as in nontransgenic littermates. AOX and control animals fed on ketogenic diet both showed wide variance in weight gain. Analysis of the gut microbiome in stool revealed a strong correlation with diet, rather than with genotype. The microbiome of the most and least obese outliers reared on the ketogenic diet showed no consistent trends compared with animals of normal body weight. We conclude that AOX expression in mice does not modify physiological responses to extreme diets.

## Introduction

Mitochondria represent the hub of energy metabolism in virtually all nonphotosynthetic eukaryotes, being responsible for the reoxidation of the primary electron carriers via the respiratory chain which, in turn, is coupled to ATP synthesis. In mammals, the energetic balance of this system of oxidative phosphorylation (OXPHOS) is modified in specific tissues and physiological conditions, notably in brown adipose tissue in the cold, where a large proportion of the energy yield from mitochondrial respiration is released as heat. Repurposing of the mitochondria for heat production involves a natural bypass of ATP synthase, in which protons extruded into the mitochondrial intermembrane space during electron transfer in the respiratory chain complexes are channeled back thermogenically into the mitochondrial matrix by uncoupler protein 1, UCP1 (reviewed by Chouchani et al. 2019).

Most eukaryotes, with the exception of vertebrates and some other fast‐moving animals, possess an alternative enzymatic machinery for mitochondrial respiration, bypassing specific OXPHOS complexes (Schertl and Braun, 2014; Rogov and Zvyagilskaya, 2015; Del‐Saz et al. 2018; McDonald and Gospodaryov 2018; Antos‐Krzeminska and Jarmuszkiewicz 2019). These alternative respiratory chain enzymes are also thermogenic, as famously documented in plants (Meeuse 1975; Wagner et al. 2008). The alternative respiratory enzymes include the alternative oxidase AOX, which bypasses the proton‐motive steps of respiratory complexes III and IV. When enzymatically active, AOX catalyzes the passage of electrons directly from ubiquinol to molecular oxygen in a non proton‐motive reaction, which is thus thermogenic. In those organisms endowed with it, AOX is able to buffer stress in the mitochondrial respiratory chain resulting from genetic or physicochemical (e.g., oxidative) damage, overload, or inhibition by toxins (see recent reviews by: Del‐Saz et al. 2018, McDonald and Gospodaryov 2018). To ensure that its presence does not disturb electron flow in the respiratory chain under normal physiological conditions, it is only active when its electron‐donor substrate, ubiquinol, accumulates to abnormally high levels, indicating inhibition of the respiratory chain at a downstream step (Dry et al. 1989; Hoefnagel and Wiskich, 1998; Dogan et al. 2018). Various additional metabolic effectors, such as pyruvate, other organic acids and nucleotides can enable or potentiate the activation of AOX in plants, fungi, and protists, although little is known of how AOX is regulated in animals (McDonald and Gospodaryov 2018; Saari et al. 2019). The evolutionary loss of AOX from vertebrates is a matter for speculation. If the enzyme were to become active under stress conditions, for example, in fight‐or‐flight situations or under starvation, it could impair survival. Alternatively, while plants and sessile invertebrates are unable to escape from a stressful environment, vertebrates can simply move somewhere else.

Based on the foregoing, we reasoned that the introduction of AOX into humans or human cells might be of therapeutic benefit in diseases associated with mitochondrial respiratory chain dysfunction (El‐Khoury et al. 2014). Moreover, this approach provides a useful tool to probe the involvement of the mitochondrial respiratory chain in diverse pathological models. We therefore introduced AOX from the tunicate *Ciona intestinalis*, the organism phylogenetically closest to humans which has retained the AOX gene, into human cells. Initially this was carried out in the HEK293T cell line (Hakkaart et al., 2006). Subsequently AOX was introduced into cells with OXPHOS defects arising from RNAi treatment or from disease‐causing mutations (Dassa et al., 2009). In these contexts, AOX was efficiently expressed, targeted to mitochondria, shown to be enzymatically active and to mitigate defects arising from OXPHOS impairment, such as metabolic acidosis, oxidative stress or poor growth on low‐glucose media.

Subsequently, *Ciona* AOX was introduced into model organisms. *Drosophila* ubiquitously expressing AOX was found to be viable, and to experience only minor effects on development and physiology under standard laboratory conditions, compared with controls. Similarly, two different lines of mice ubiquitously expressing AOX (El‐Khoury et al. 2013; Szibor et al. 2017) showed no significant deviations from controls, even when over 350 physiological, behavioral and metabolic parameters were measured. Nevertheless, AOX was able to confer protection against various kinds of physiological stress, ranging from exposure to respiratory poisons such as cyanide or antimycin A (Fernandez‐Ayala et al. 2009; El‐Khoury et al. 2013; Szibor et al. 2017), genetically engineered OXPHOS deficiency (Kemppainen et al. 2014; Rajendran et al. 2019) pathological models related to oxidative, proteotoxic, nutritional, and proinflammatory stress (Fernandez‐Ayala et al. 2009; Humphrey et al. 2012; El‐Khoury et al. 2016; Mills et al. 2016; Giordano et al. 2018), or disturbed nuclear‐receptor and Jun‐kinase signaling (Andjelković et al., 2016; Andjelković et al., 2018).

Nevertheless, flies expressing AOX showed a slightly exaggerated weight loss in the early weeks of adult life compared with controls (Fernandez‐Ayala et al. 2009), suggesting the possibility that AOX may become partially active at that stage, compromising the efficiency with which useful energy in the form of ATP is recovered from food substrates. Furthermore, under conditions of nutritional stress, AOX‐expressing flies were impaired in their ability to complete development on low‐nutrient medium containing only glucose and yeast (Saari et al. 2019).

In mammalian models, nutritional limitation, such as by caloric restriction, has been successfully used to study aging, but more complex dietary experiments to identify specific nutritional requirements are problematic. This is due, for example, to the large number of traits influenced by diet, the diversity of inbred strains, and the effects that may be mediated by the microbiome, especially in pathogen‐free facilities (National Research Council (US) Subcommittee on Laboratory Animal Nutrition, 1995). An alternative approach, which has been widely adopted in the use of mammalian models to study obesity and metabolic disease, is to feed animals on a diet containing a high content of fats and/or a low content of carbohydrates. Mice of susceptible strains (West et al. 1992; Champy et al. 2008) fed a high‐fat (HF) diet show increased weight and many physiological detriments, including cardiac dysfunction (Khan et al. 2010; Dirkx et al. 2011; Shuai et al. 2019), compared with those fed a control (chow) diet. Susceptible mice, such as of strain C57BL/6J, also show cardiac impairment (Nilsson et al. 2016) when fed a high‐fat low‐carbohydrate diet usually described as ketogenic, since ketone bodies produced in the liver from dietary fat become the main metabolic fuel for organs normally dependent on sugars, such as the brain. When fed a ketogenic diet C57BL/6J mice have been reported to exhibit slower weight gain than controls when fed *ad libitum* (Thio et al. 2006) or with controlled isocaloric food intake (Kennedy et al. 2007). However, in a separate study no overall weight difference from controls was observed, despite altered body composition, with increased fat but decreased lean mass (Nilsson et al. 2016). Conversely, mice exposed for long periods to ketogenic diet have been shown to gain weight compared with chow‐fed controls (Ahola‐Erkkilä et al. 2010). Ketogenic diet has also been shown to lead to changes in the composition of the gut microbiome (Ma et al. 2018), which was suggested to minimize inflammation.

The findings with AOX‐expressing flies under nutritional limitation led us to hypothesize that partial activation of AOX could modify energetic balance in the mice in ways that would alter the response to dietary stress. Mouse models with increased energy expenditure typically have a lean phenotype, with decreased weight gain in fat‐rich diets. Examples are knockout of the Maf1 regulator of Pol III transcription (Willis et al. 2018), treatment with OXPHOS uncouplers (Goldgof et al. 2014; Kalinovitch and Shabalina 2015; Suzuki et al, 2015) and genetic modifications that increase sympatho‐adrenal tone (Ruohonen et al. 2018). Thus, our expectation was that AOX would also have this effect in mice. Such an outcome could be of considerable importance in regard to the future development of AOX‐based therapy and its possible utility in combating obesity, impaired glucose tolerance and related metabolic disorders, as suggested by the findings in *Drosophila*.

## Methods

### Laboratory animals and procedures

Wild‐type mice (C57BL6/JOlaHsd, RRID:MGI:5658456) were purchased from Envigo. Mice globally expressing *C. intestinalis* AOX inserted at the *Rosa26* locus (*Rosa26^Aox^*), described by Szibor et al. (2017), were backcrossed into the C57BL6/JOlaHsd background over >10 generations before the start of the experiment. To minimize genetic drift, the stock of wild‐type mice was replaced annually from the same vendor. All animals were treated according to the regulation of the Finnish animal welfare board (ELLA), under ethical permit ESAVI/8766/04.10.07/2015. Mice were housed in a humidity and temperature‐regulated animal facility with food and water provided *ad libitum* and a 12‐hour light/dark cycle. They were euthanized at the end of the experiment by cervical dislocation. Humane end points were followed, as described in the ethical permit. Mouse echocardiography by ultrasound (Vevo 2100, RRID:SCR_015816) and body weight measurement were performed as previously described (Szibor et al. 2017). Mouse body composition was measured using DEXA imaging system (GE LUNAR PIXImus2 imager) according to the guidelines of EMPReSS (RRID:SCR_003087). Before imaging, animals were anesthetized with a 1:1 mixture of pentobarbital (Orion Pharma, 100 mg/kg of body weight) and lidocaine (Orion Pharma, 16 mg/kg of body weight). Blood droplets were collected from the tail vein to measure glucose, lactate, and ketone‐body concentrations using Freestyle Precision (Abbot Laboratories, USA), Lactate Pro (Arkay, Kyoto, Japan), and Freestyle Precision metering instruments, respectively, according to manufacturer's instructions. After imaging, mice were sacrificed by cervical dislocation.

### Diet manipulations

Diets were purchased from Envigo, and were sterilized by irradiation. Male littermates were randomized into different diet groups and the diets were introduced 8 weeks after birth, this delay ensuring that they were already accustomed to solid food. Animals were caged in littermate groups (2–5 per cage) for the initial diet manipulation experiment. For the follow‐up experiment on ketogenic diet only, mice were caged individually in a different animal facility. Diets were standard chow (Teklad 2918, Teklad Global 18% Protein Rodent Diet, providing 24% of total calories from protein, 18% from fat, and 58% from carbohydrates); high‐fat (Teklad Custom Diet TD.06414, providing approximately 60% of total calories from fat, comprising 37% saturated, 47% monounsaturated, and 16% polyunsaturated) and ketogenic (Teklad Custom Diet TD.96355, providing 90% of total calories from fat with almost none (~0.5%) from carbohydrates).

### Genotyping

All animals used in the experiments were PCR‐genotyped for the *Rosa26* locus and AOX transgene prior to and at the endpoint of the experiments, using ear‐punch and tail‐tip DNA, respectively, as described previously (Szibor et al. 2017). Animals were thereby assigned either as wild‐type at *Rosa26* or hemizygous for AOX.

### Gut microbiome analysis

Animal droppings were collected from mice for microbiome analysis. DNA extraction, PCR and sequencing and analysis were performed as described (Salava et al. 2017), except that the primers used for PCR were as follows (all 5´ to 3´): Illum_341F_1,

ACACTCTTTCCCTACACGACGCTCTTCCGATCTCCTACGGGNGGCWGCAG; Illum_341F_2, ACACTCTTTCCCTACACGACGCTCTTCCGATCTgtCCTACGGGNGGCWGCAG;

Illum_341F_3,

ACACTCTTTCCCTACACGACGCTCTTCCGATCTagagCCTACGGGNGGCWGCAG;

Illum_341F_4,

ACACTCTTTCCCTACACGACGCTCTTCCGATCTtagtgtCCTACGGGNGGCWGCAG.

and Illum_785R_1,

GTGACTGGAGTTCAGACGTGTGCTCTTCCGATCTGACTACHVGGGTATCTAATCC

Illum_785R_2,

GTGACTGGAGTTCAGACGTGTGCTCTTCCGATCTaGACTACHVGGGTATCTAATCC;

Illum_785R_3,

GTGACTGGAGTTCAGACGTGTGCTCTTCCGATCTtctGACTACHVGGGTATCTAATCC;

Illum_785R_4,

GTGACTGGAGTTCAGACGTGTGCTCTTCCGATCTctgagtgGACTACHVGGGTATCTAATCC.

Sample fastq files were processed with cutadapt (RRID:SCR_011841, v.1.8.3), to remove primers and low‐quality ends of the sequences, with parameter ‐q 28, and reads that were shorter than 180 bp were removed with cutadapt parameter ‐m 180. Mothur (RRID:SCR_011947, v. 1.40.2) was used for further sequence processing following the operating procedure of the standard Illumina MiSeq System (RRID:SCR_016379), and mothur paired‐end sequences were joined to contigs and further processed by removing chimeras using the VSEARCH algorithm (Rognes et al. 2016), discarding singleton sequences, clustering sequences into operational taxonomic units (OTUs) at ≥97% similarity, and finally assigning taxonomy to OTUs. Data visualization (relative abundance) and differential abundance analyses were performed in RStudio (RRID:SCR_000432, R version 3.4.4), and the phyloseq package (RRID:SCR_013080, v. 1.20.0) was used for managing taxonomy and OTU data in R. Finally, data were refiltered to include only taxa having> 4 reads in> 2 samples, prior to differential abundance analyses with DESeq2 (RRID:SCR_015687, v. 1.16.1).

## Statistics

All data regarding total body weight, body composition, blood metabolite levels, and cardiac functions by echocardiography are reported as mean ± SD. Statistical analyses of all data, except gut microbiome analysis, were performed using GraphPad Prism (RRID:SCR_002798). Differences in body weight, body composition, blood metabolites, and cardiac parameters were analyzed using two‐way ANOVA followed, where appropriate, by Tukey’s multiple comparisons test. *P* values ≤ 0.05 were considered statistically significant. Significantly different data classes are denoted by lower‐case letters in the figures, with relevant conclusions highlighted in the text. Differential abundance analysis of gut microbiome taxa was conducted with DESeq2 (RRID:SCR_015687, v. 1.16.1). Details of statistical analyses, including all *p* values, are shown in supplementary tables, as referenced in each figure legend.

## Results

### AOX does not affect patterns of weight gain in different diets

**Figure 1:**
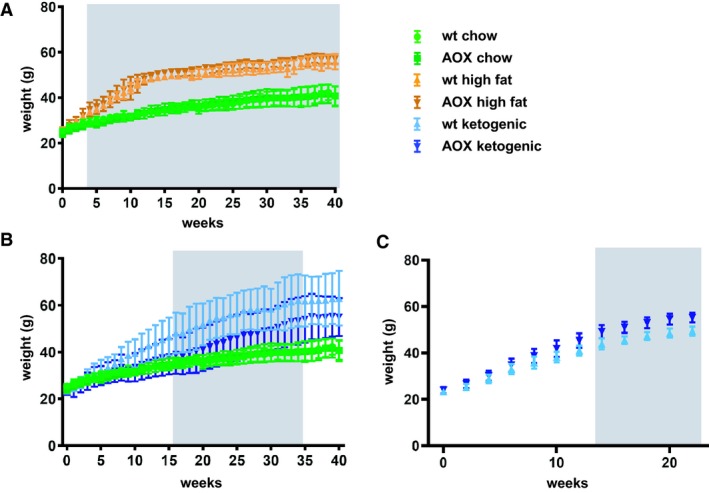
AOX expression has minimal effects on total body weight of mice fed on fat‐rich diets. (A, B) Body weight (means ± SD) of wild‐type (wt) and AOX‐expressing male mice fed with either standard chow, high‐fat, or ketogenic diets, as indicated. Highlighted gray areas indicate statistical significance (*P* < 0.05, two‐way ANOVA followed by Tukey’s multiple comparisons analysis *n* ≥ 6 for all groups) (A) between diets but not genotypes, (B) between genotypes. Note that, for clarity, a single data set from chow‐fed animals is shown in both (A) and (B), alongside data, respectively, from (A) high‐fat diet‐fed and (B) ketogenic diet‐fed animals. (C) represents data from a separate experiment (ketogenic diet only), in which animals were caged individually, n ≥ 4 for all groups, gray area denoting statistical significance between genotypes, otherwise similar nomenclature and statistical analysis as in (A, B). See Tables S1 for full details of statistical analysis.

We initially studied groups of AOX mice and nontransgenic male littermates reared on three different diets: standard chow, high‐fat diet (60% of total energy from fats) and ketogenic diet (90% of total energy from fats, with almost no carbohydrates). Mice were transferred to these diets at 8 weeks of age, that is, approximately 4 weeks after weaning, prior to which they had been maintained on standard chow. Note that, in this first experiment, littermates were housed together, regardless of genotype. For convenience, the effects of high‐fat diet and of ketogenic diet on total body weight are shown separately (Fig. 1A, 1), although a single set of chow‐fed controls was used in the experiment. Three conclusions emerge. First, animals of both genotypes showed accelerated weight gain on high‐fat diet compared with standard chow during the first 10–12 weeks of the experiment. Thereafter, the rate of weight gain was essentially the same for animals fed on high‐fat as for chow diets. Second, there was no significant difference between genotypes on either high‐fat or chow diet. Third, we found that wild‐type animals reared on ketogenic diet gained weight at the same rate as those fed standard chow during the first 10 weeks of the experiment. Thereafter they began to gain weight faster than chow‐fed controls. AOX mice also gained weight on ketogenic diet after 10 weeks. In addition, AOX and wild‐type mice, despite being in the same inbred background, showed considerable phenotypic variation in the extent of weight gain. Moreover, the two groups were significantly different from each other between weeks 16‐35, with AOX mice showing slightly less weight gain than wild‐type littermates. Because of the wide variation observed on ketogenic diet, this part of the experiment was repeated using a different caging protocol, where all animals were reared in separate cages, so as to minimize any behavioral issues. This produced a different result (Fig. 1C) in which weight variation within the groups was no longer evident. However, the amount of weight gain was once more slightly different between the two genotypes, except in this case in the opposite direction, with AOX mice showing slightly greater weight gain on ketogenic diet than controls. Combined with the large variation seen in the first experiment, this leads us to infer that there was no systematic difference in the pattern of weight gain on ketogenic diet between the genotypes.

### AOX has minimal effects on the changes in body composition caused by fat‐rich diets

**Figure 2:**
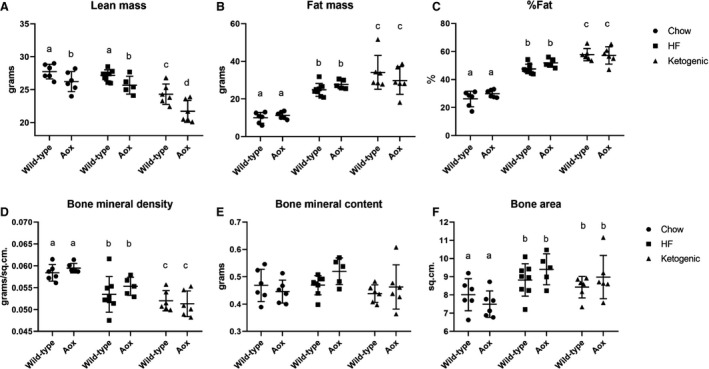
AOX expression has minimal effects on body composition of mice fed on high‐fat or ketogenic diet. Dual‐energy X‐ray absorptiometry (DEXA) analysis of bone, muscle, and fat parameters as indicated, on 48‐week‐old mice (*n* ≥ 6 for all groups) of the indicated genotypes, fed on standard chow, high‐fat (HF), or ketogenic diet as shown. Two‐way ANOVA followed by Tukey’s multiple comparison analysis, with lower‐case letters denoting significantly different data groups in each panel. Note that fat percentage (C) is extrapolated from lean mass (A) and fat mass (B). Similarly, bone mineral density (D) is extrapolated from bone mineral content (E) and bone area (F). See Tables S2 for full details of statistical analysis, including all *P* values.

**Figure 3:**
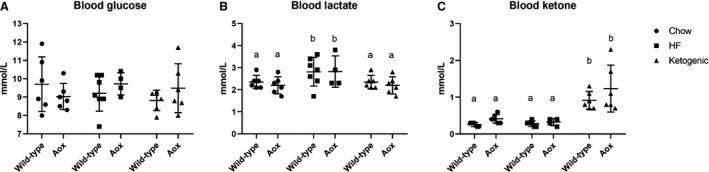
AOX expression has minimal effects on diet‐dependent changes to key blood metabolites. (A) Glucose, (B) lactate, and (C) ketone‐body concentrations measured from tail‐vein blood of 48‐week‐old mice (*n* ≥ 4 for all groups) of the indicated genotypes, fed on standard chow, high‐fat (HF), or ketogenic diet, as shown. Two‐way ANOVA followed, where appropriate, by Tukey’s multiple comparison analysis, with lower‐case letters denoting significantly different groups in each panel. See Tables S3 for full details of statistical analysis, including all *P* values.

Both of the fat‐rich diets produced significant changes in body composition, measured at 48 weeks of age, immediately before sacrifice. These were broadly similar between the genotypes (Fig. 2), although the AOX mice showed a slight but significantly decreased lean mass compared with wild‐type controls, in all diets (Fig. 2A). Both high‐fat and ketogenic diet resulted in increased fat mass (Fig. 2B), with ketogenic diet also producing decreased lean mass (Fig. 2A), such that the overall increase in fat percentage (Fig. 2C) was greatest for the animals reared on ketogenic diet. Bone mineral density (Fig. 2D) was decreased by high‐fat and even more so by ketogenic diet, due mainly to alterations in bone area (Fig. 2F), while bone mineral content were more variable and did not reach significance (Fig. 2E). Measurements of blood glucose (Fig. 3A) showed no significant changes according to diet or genotype, while lactate (Fig. 3B) was slightly (but significantly) increased by high‐fat diet, and ketone bodies were elevated, as expected, by ketogenic diet (Fig. 3C).

### AOX does not modify the impaired heart performance caused by ketogenic diet

**Figure 4:**
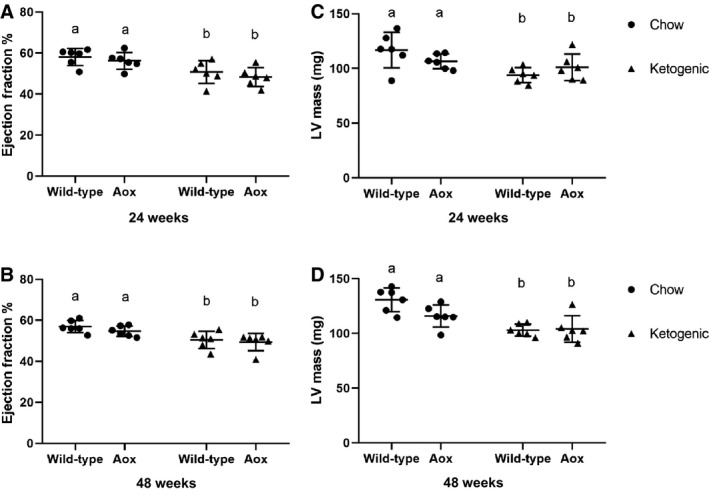
AOX expression has minimal effects on ketogenic diet‐dependent changes in cardiac parameters. Echocardiography (means ± SD, *n* ≥ 6 for all groups) from 24‐ and 48‐week‐old mice as shown. (A, C) ejection fraction; (B, D) left ventricular mass. Two‐way ANOVA, with lower‐case letters denoting significantly different data classes in each panel. See Tables S4 for full details of statistical analysis, including all *P* values

In accord with previous literature, we observed impaired cardiac function in mice reared on ketogenic diet (Fig. 4). There was a trend towards decreased ejection fraction after 24 weeks on the diet, and this was maintained and was statistically significant in both genotypes by 48 weeks. At the latter time point there was also decreased left ventricular mass. However, we detected no differences between AOX and control mice.

### Microbiome changes follow diet more closely than genotype or total weight

**Figure 5:**
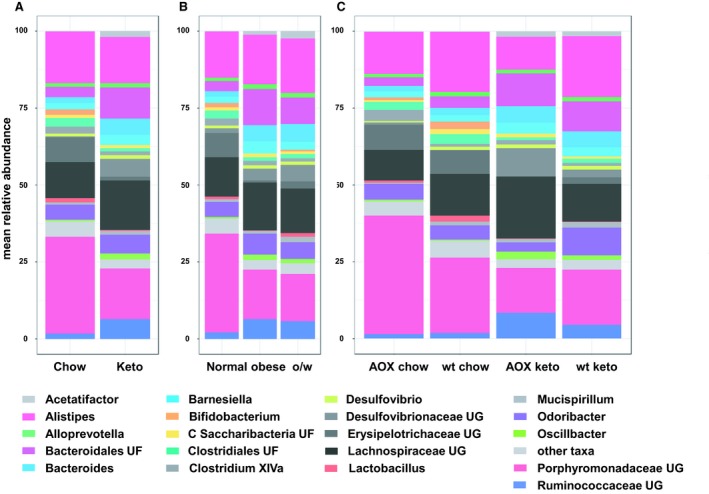
Microbiome changes associated with genotype, diet, and weight. Distribution of top 20 gut microbial taxa by abundance. (A) Comparing animals reared on chow versus ketogenic diet, (B) comparing different body weight groups by quartiles: normal weight (first two quartiles, <45.1 g, overweight (o/w, third quartile, 45.2 to 55.8 g) and obese (fourth quartile,> 55.8 g), (C) comparing animals by both genotype (wild‐type, wt, or AOX‐expressing) and diet. Taxa denoted by genus, or family: UF – unidentified family, UG – unidentified genus, C – candidate phylum. For further details and statistical analysis see Tables [Link], [Link], [Link], [Link]. n = 6 for all groups.

The wide variation in total body weight seen in animals of both genotypes reared on ketogenic diet suggests an influence from an uncontrolled external variable. One obvious such variable could be the population of gut microbes, which has previously been reported to show systematic variation according to diet, although it is unclear whether such changes are adaptive or maladaptive. To address this issue, we sampled the microbiome of individual animals via a high‐throughput sequencing approach, then looked for statistically significant differences according to genotype, diet, or total body weight (Fig. 5, Tables [Link], [Link], [Link], [Link]). There were few significantly different genera and no significantly different families between genotypes (Tables S5), and none of the top 20 taxonomic units by abundance was changed. Between diets (chow vs. ketogenic), there were a number of systematic differences (Tables S6, Fig. 5A), most of which were shared between the genotypes (Tables S7, Fig. 5C). When considering the animals purely according to weight category (Fig. 5B, Tables S8), we looked for significant changes between animals of normal weight range (median and below) and the next quartile (overweight), then asked which of them were altered further in the obese group (heaviest quartile), whether in the same or opposite direction. Despite some apparent trends (Fig. 5B), we found no taxonomic categories that showed significant change in both comparisons (Table S8).

## Discussion

Previous studies showed that mice ubiquitously expressing AOX (El‐Khoury et al. 2013; Szibor et al. 2017) exhibited no physiological deviations from controls, when studied under standard laboratory conditions. However, the relevant assays were conducted at a single, early lifetime point, under nonstressful conditions. In contrast, the present investigation aimed to determine the physiological effects of long‐term dietary stress on the AOX mice which, based on findings in *Drosophila*, were expected to reveal clear differences from controls. Unexpectedly, the differences observed were minimal. Total body weight, overall body composition and, in the case of ketogenic diet, impaired cardiac function, were not systematically altered by AOX. AOX also had only minimal effects on the gut microbiome. The results stand in contrast to studies in disease models, where AOX has been shown to have clear‐cut effects that could be either beneficial (Mills et al. 2016; Rajendran et al. 2019) or detrimental (Dogan et al. 2018) to survival.

### Conditions for AOX activation

The simplest explanation for these findings is that AOX remains enzymatically inert, even under the rather extreme conditions of ketogenic diet. AOX is regulated by the reduction state of its substrate ubiquinol, being effectively outcompeted by OXPHOS complex III (ubiquinol:cytochrome *c* oxidoreductase) except when the reduced quinone reaches a substantial proportion of the total quinone pool (Dry et al. 1989; Hoefnagel and Wiskich, 1998); this has been verified for the *Ciona* enzyme when expressed in mouse, under conditions of complex IV (cytochrome *c* oxidase) deficiency (Dogan et al. 2018). In plants, AOX is also regulated by pyruvate (Hoefnagel and Wiskich 1998; Sierra‐Campos et al. 2009; Carré et al. 2011; Chen et al. 2015) which affects its quaternary structure, and in diverse taxa by other organic acids (Siedow and Umbach 2000; Selinski et al. 2018), nucleotides (Sierra‐Campos et al. 2009; Selinski et al. 2018), and calcium (Mariano et al. 2005; Zalutskaya et al. 2015). Despite the metabolic disturbances resulting from fat‐rich diets (Barrington et al. 2017; Fujisaka et al. 2018; Hassan et al. 2018; Lee et al. 2018; Showalter et al. 2018; Tomášová et al. 2018; Gu et al. 2019), including the production of ketone bodies and many compounds included within or related to the aforementioned categories, it was not possible to determine whether or not AOX was enzymatically active *in vivo*, during the present study. This would require the development of entirely novel, noninvasive methods. If the enzyme were shown to have become activated in mice fed a fat‐rich diet, its effects on energy yield would have to have been compensated by increased food intake, absorption or processing, or drastically curtailed activity, to have produced the observed end result of no effect on weight gain.

Our findings imply that the stressful effects of fat‐rich diets are not mediated by respiratory chain impairment at the level of complexes III or IV. Moreover, the fact that blood glucose and lactate levels are not affected by diet or by AOX (Fig. 3) argues against an upstream effect at complex I. To unravel these possibilities will require further testing using controlled feeding and continuous metabolic monitoring, including tests of glucose tolerance. In contrast to our findings with AOX, a futile cycle of energy wastage has been proposed to underlie the resistance to high‐fat diet‐induced weight gain of *Maf1* knockout mice, which show elevated RNA polymerase III‐dependent transcription (Willis et al. 2018).

### Microbiome changes do not correlate with extent of weight gain on ketogenic diet

Because food intake may be an issue, as discussed below, we considered the microbiome data in regard to multiple, potentially interacting factors: diet and genotype, (Table S7, Fig. 5C), and weight only (Tables S8, Fig. 5B). Our finding that there were no trends in the composition of the intestinal microbiota of highly obese mice compared with other groups, contrasts with previous findings which identified a correlation between changes in the microbiome and intestinal energy balance (Turnbaugh et al. 2006; Wichmann et al. 2013). Although it would be interesting to compare wild‐type chow‐fed animals with AOX animals fed on ketogenic diet that were in the lowest weight gain group, the number of animals studied was insufficient for this further stratification to be reliably meaningful. This could be investigated in a follow‐up experiment, although it would be hard to justify, given the absence of any clear signal evident in the present study.

A previous study (Ma et al. 2018) reported specific changes in the microbiome produced by ketogenic diet, with increased abundance of taxa considered “beneficial” (*Akkermansia muciniphila*, *Lactobacillus*), and decreases in *Desulfovibrio* and *Turicibacter*, regarded as proinflammatory. One of these changes was seen in the present study, namely the decrease in *Turicibacter* (Tables S6), while *Lactobacillus* and *Desulfovibrio* showed opposite changes to those reported by Ma et al. (2018), and *A. muciniphila* was unchanged. The diet‐dependent decrease in *Turicibacter* was seen in both genotypes (Tables S7), but *Turicibacter* was already low in AOX mice fed on chow diet, as was *Desulfovibrio*.

In a previous study using a similar approach, the microbiome of the most obese (“obesity‐prone”) versus the less obese (“obesity‐resistant”) wild‐type mice fed a high‐fat diet was found to differ (Gu et al. 2019). However, this experiment was performed on low numbers of mice (*n* = 3) and shows no coherence with our own findings using ketogenic diet. Note, however, that different primer pairs from those employed by Gu et al. (2019) were used in our study. Other authors have reported a correlation between metabolic disturbance, obesity, dietary fat, and the microbiome (Ke et al. 2019), as well as with behavior (Hassan et al. 2018). It remains unresolved whether the microbiome changes are the result of variations in these other parameters or are instrumental in producing them.

Mice fed either of two antioxidants, Tempol (Jiang et al. 2015; Cai et al. 2016), or N‐acetyl cysteine (NAC Falach‐Malik et al. 2016; Zheng et al. 2019), showed alleviation of diet‐induced phenotypes, together with alterations in the microbiome. Since AOX has previously been inferred to be functionally equivalent to an antioxidant in *Drosophila* (Fernandez‐Ayala et al. 2009; Sanz et al. 2010), we looked for any changes in the microbiome of AOX mice that were similar to those reported in the literature for NAC‐treated mice. Two of the most striking differences produced by NAC, especially in high‐fat diet, were increased amounts of *Bifidobacterium* and *Lactobacillus* (Zheng et al. 2019). However, *Bifidobacterium*, like *Lactobacillus*, was actually decreased in AOX expressors compared with control mice on chow diet (Tables S7), and was not significantly different between the genotypes on ketogenic diet (Tables S7) or when considered without regard to diet (Tables S5).

### Other possible causes of weight variation in ketogenic diet

In the first experiment, the large variation in total body weight on ketogenic diet increased with age, but was already a clear trend, if not yet significant, after 10–12 weeks (Fig. 1B). In the second experiment, the variation within the groups was modest even at 20 weeks, at which point the experiment was terminated. A reasonable assumption is that differences in the caging system (littermate groups vs. individual caging) are likely to underlie the different outcomes. The wide variation in weight gain is logically a reflection of differences in energy expenditure and/or in feeding, for which behavioral interactions between males caged together are a logical explanation. Mice are highly territorial, and certain males can retain power over food intake of others (Ellacott et al. 2010). Since the outliers in the experiment (i.e., the most and least obese animals) were from multiple cages, this seems a more likely explanation than an unidentified external stressor, such as noise, vibration, or chemical exposure. In practice, it may be impossible to eliminate all possible sources of environmentally induced behavioral variation (Crabbe et al. 1999). Our study illuminates the value of repeating such experiments in a different environment before attempting to draw firm conclusions.

Caging male mice in groups (first experiment, Fig1B) or individually (second experiment, Fig. 1C) potentially has different behavioral consequences, either of which can be stress‐related. Whereas individual caging is considered a paradigm for social isolation (Võikar et al. 2005), animals caged in groups develop social hierarchies that can influence feeding behavior and lead to divergent patterns of weight gain, which has been extensively documented in rats (Tamashiro et al. 2007a, 2007b) as well as mice (Goto and Toyoda 2015; Razzoli et al. 2017; Keenan et al. 2018).

Genetic heterogeneity at loci other than the AOX insert is an extremely unlikely factor to explain the wide variation in weight gain, since the animals had all been back crossed over >10 generations to a pure background strain, with fresh breeding animals sourced annually. In an unlikely scenario, if a relevant allele were present in just one or a few animals used for backcrossing, it could create undetected genetic variation. However, it is then hard to account for the fact that variation in weight gain occurred in just one of two sequential experiments.

### Implications for future therapeutic uses of AOX

One of the underlying reasons we conducted this study was to determine if AOX, proposed as a future therapy for many different diseases (El‐Khoury et al., 2014), would interact with “stressful” diets with high‐fat content to produce undesired physiological effects. In such a case, any therapeutic use would have to be conducted in combination with a strict dietary regime. Conversely, the prior data from *Drosophila* (Fernandez‐Ayala et al. 2009) suggested that AOX expression might, under some circumstances, promote weight loss or mitigate diet‐induced weight gain and other detriments, via futile expenditure of energy. It was suggested that this might even be a viable treatment for obesity and other metabolic diseases. The results of the study would seem to exclude both propositions, at least as far as the C57BL6 mouse is concerned. However, extrapolating the findings to humans obviously must await future clinical trials, if AOX passes other safety and efficacy criteria.

## Conflict of interests

MSz is a shareholder in a startup company aiming to develop therapeutics based on AOX. The authors declare no other competing interests.

## Supporting information




**Table S1** **.** Statistical analyses for Figure 1

**Table S2** **.** Statistical analyses for Figure 2

**Table S3** **.** Statistical analyses for Figure 3

**Table S4** **.** Statistical analyses for Figure 4

**Table S5** **.** Statistical analyses of microbiome data, compared by genotype only (AOX versis wild‐type (Wt)

**Table S6** **.** Statistical analyses of microbiome data, compared by diet only (Ketogenic versus Chow)

**Table S7** **.** Statistical analyses of microbiome data, compared by diet and genotype

**Table S8** **.** Statistical analyses of microbiome data, compared by weight groups (normal, over‐weight, obese)
